# Periapical healing in endodontic retreatment: A comparative study of nano-hydroxyapatite with and without PRF

**DOI:** 10.6026/973206300211195

**Published:** 2025-05-31

**Authors:** Shelly Singh, Jnana Ranjan Swain, Megha Gugnani, Ashtha Arya, Ranjita Singh Baghel, Samra Shafique

**Affiliations:** 1 Department of Dental Surgery, School of Dental Medicine, University of Colorado, USA; 2 Department of Pedodontics and Preventive Dentistry, Institute of Dental Sciences, Siksha O Anusandhan (Deemed to be University), Bhubaneswar, Odisha, India; 3 Department of Conservative and Endodontics, Yamuna Institute of Dental Sciences and Research, Gadhauli, Yamunanagar, Haryana, India; 4 Department of Conservative Dentistry and Endodontics, SGT Dental College, Hospital and Research Institute, SGT University, Gurugram, Haryana- 122505, India; 5 Department of Periodontics, Index Institute of Dental Sciences, Indore, Madhya Pradesh, India; 6 Department of Conservative Dentistry and Endodontics, Sardar Patel Postgraduate Institute of Dental and Medical Sciences, Raibareli Rd, Utrathia, Vrindavan Colony, Lucknow, Uttar Pradesh 226025, India

**Keywords:** Periapical pathosis, bone healing, nano-hydroxyapatite, Platelet-Rich Fibrin (PRF), retreatment, nano-hydroxyapatite (nHA)

## Abstract

The efficacy of nano-hydroxyapatite (nHA) alone and in combination with platelet-rich fibrin (PRF) in enhancing periapical bone healing following endodontic retreatment is of interest. Thirty-two patients with failed endodontic treatment in anterior teeth were divided into two groups. After retreatment, apicoectomy and root-end filling with mineral trioxide aggregate (MTA) were performed. Group 1 received nHA alone, while Group 2 received a combination of nHA and PRF in the periapical cavity. Follow-ups at 1, 3, and 6 months showed significant lesion size reduction in both groups, with faster healing in the nHA+PRF group at 1 month, but no long-term differences.

## Background:

Periapical lesions are one of the common pathological conditions affecting periradicular tissues [1]. The microbial invasion and subsequent infection of the canal systems of a root play a decisive role in the initiation and progression of periapical lesions [2]. Periapical lesions are mostly classified as radicular cysts, dental granulomas, or abscesses [3, 4]. The primary goal of all endodontic procedures, particularly cleaning and shaping, is to remove necrotic tissue and eradicate infectious bacteria [5]. The success of root canal treatment relies on complete periapical healing, which is commonly accomplished through non-surgical endodontic procedures [6]. However, in cases where symptoms persist and infection lingers despite non-surgical treatment. In such situations, periradicular surgery may be required to excise the pathological tissues and eliminate sources of irritation. This surgical procedure focuses on eradicating any remaining infection and promoting the regeneration of healthy periapical tissue [7]. One effective method involves utilizing Platelet-Rich Fibrin (PRF), which has demonstrated success in promoting bone regeneration when combined with osseous grafts for the treatment of periapical defects. PRF stimulates a cascade of healing events, including cell proliferation, collagen synthesis and angiogenesis, which contribute to enhanced tissue regeneration. When used in conjunction with bone grafts, PRF has been shown to result in excellent osseous defect fill, as evidenced by radiographic assessment, making it a valuable tool in accelerating bone healing and improving outcomes in periapical and other oral surgical procedures [8]. Several bone graft materials, including bioactive calcium phosphate ceramics, have been utilized to improve osseous healing. These materials, including hydroxyapatite (HA) and tricalcium phosphate (TCP), represent the largest family of alloplastic grafts. Both HA and TCP have been employed extensively to promote bone regeneration and enhance bone fill following periapical surgery. Their bioactive properties encourage osteo-conduction, allowing new bone to grow along the scaffold and in some cases; they can even stimulate osteo-induction, further supporting the healing process. As a result, these calcium phosphate ceramics are valuable adjuncts in the treatment of periapical pathologies [9, 10, 11- 12]. Recently, nanotechnology has been integrated into dentistry to design and develop materials, devices and systems by manipulating matter at the nanometer scale, including atoms, molecules and supramolecular structures. This innovative approach enables the creation of dental materials with superior properties, such as enhanced strength, biocompatibility and functionality. By harnessing the unique attributes of nanomaterials, such as their increased surface area and reactivity, this technology holds great potential for significantly improving clinical outcomes [13]. Nanohydroxyapatite (nHA) exhibits unique properties due to its small size and large surface area, allowing for improved interactions with biological tissues. In this study, nHA was used alone and in combination with Platelet-Rich Fibrin (PRF) to promote osseous healing in the periapical region. Therefore, it is of interest to assess the potential benefits of nHA and its synergy with PRF in enhancing bone regeneration following periapical surgery.

## Methods and Methodology:

The selection criteria for patient inclusion were as follows:

[1] Patients willing to participate in the study and attend regular follow-up appointments.

[2] Patients without chronic conditions that could interfere with anesthesia or impede healing.

[3] Patients presenting with an anterior necrotic tooth with a periapical lesion of at least 5 millimeters in diameter and a history of unsuccessful endodontic treatment.

[4] Patients who provided informed consent before participation.

[5] Measures were implemented to protect the privacy and security of patient's personal and health information. All data remained confidential and the study received approval from the ethics committee.

## Exclusion criteria:

[1] Patients who received recent head and neck radiation or chemotherapy within the past year, as these treatments can impair healing and complicate the outcomes of the study.

[2] Patients taking medications that may negatively impact the healing process, such as systemic steroids or anticoagulant therapy, were excluded due to their potential to interfere with bone regeneration and tissue repair.

[3] Patients with poor oral hygiene or significant periodontal problems, as these conditions could affect the healing process and complicate the study's objectives. Thirty-two patients with periapical lesions were included in this study. Periapical radiographs and CBCT scans were used to assess the size of periapical lesions (≥5 mm) in failed endodontically treated single-rooted teeth. 32 teeth were randomly divided into two groups.

## Clinic visits:

During the first visit preoperative radiographs were taken. Teeth were anesthetized (4% articaine local anesthesia) access opening was done and isolated with a rubber dam. The length of teeth was measured radio graphically and electronically using an apex locator (Mini Root ZX mini, J Morita, USA). Canals were cleaned and shaped with proTaper files. Canals were irrigated with 2.5% NaOCl and dried. Bi-antibiotic paste (metronidazole and ciprofloxacin) was placed. Access cavities were sealed with composite resin. This initial procedure aimed to disinfect and temporarily seal the canals, allowing for subsequent interventions and healing of the periapical tissues before the final filling and restoration phase. The second visit was after 10 days in which patients were re-examined. If asymptomatic, they rinsed with 0.2% chlorhexidine and proceeded to obturation and surgery. If symptomatic, canals were re-treated with NaOCl and bi-antibiotic paste for 4-6 weeks. Periapical Surgery procedures: Periapical surgery was performed under local anesthesia. A modified rectangular flap was elevated and apical curettage and resection of the root end were performed. The root-end cavity was filled by MTA.

## Division of groups:

Group 1: Nano-hydroxyapatite (nHA) powder was carefully condensed inside the body cavity to ensure the entire packing of the void space and optimal contact with the surrounding bone tissue.

Group 2: Preparation of Platelet-Rich Fibrin (PRF) Gel: A volume of 10-30 mL of the patient's blood was collected into sterile, dry Monovettes without the addition of anticoagulants before the surgical procedure. The collected blood was immediately centrifuged at 2500 revolutions per minute for 10 minutes.

## This centrifugation process separated the blood components into 3 layers:

RBCs, PRP and a fibrin clot. The fibrin clot was carefully extracted using sterile forceps and transferred into a sterile tube [14]. The nHA powder was added to the previously prepared platelet-rich fibrin (PRF) gel to mix them and then the mixture was condensed into the bony defect followed by wound suturing. Instructions were given to the patients to apply cold compresses to the surgical site for 15 minutes every hour for the first three hours postoperatively. Patients received antibiotics (Augmentin+Flagyl) and pain relievers (Bi-Profenid) for 5 days. Mouth rinses with warm saline and chlorhexidine were prescribed for 10 days. All sutures were removed after a period of 10 days. Patients were followed up at one, three and six months for clinical exams and radiographs [15]. Digital radiographs were taken using a size two sensor and film holders to allow for film positioning in a parallel position. After six months, cone-beam computed tomography (CBCT) scans were obtained to assess bone density and healing. Linear dimensional measurements in millimeters were made on pre- and post-operative radiographs to evaluate bone defect reduction [16, 17- 18]. Results data were non-normally distributed (Kolmogorov-Smirnov and Shapiro-Wilk tests). Data is presented as mean, SD, median and range. Kruskal-Wallis and Friedman tests were used for between- and within-group comparisons, respectively. Dunn's test was used for post-hoc analysis. Statistical significance was set at p<0.05. Data was analyzed using IBM SPSS statistics.

## Results:

No significant differences in lesion size were detected between the groups at preoperative, 1, 3 and 6 months (Table 1, Table 2-Table 3). In Group 1, a significant reduction in lesion size was noted over time (p < 0.001). Pairwise comparisons revealed significant decreases at 1, 1-3 and 3-6 months, indicating a continuous and sustained healing process. In Group 2, lesion size significantly decreased over time (p < 0.001), with notable reductions at 1 and 1-3 months, suggesting an initial rapid healing phase. However, from 3 to 6 months, no statistically significant change was observed, indicating that the healing process had stabilized. Percentage reduction in lesion size: calculating the percentage reduction in lesion size, it was determined as follows: [(Pre-operative size - Post-operative size) / Pre-operative size] x 100. The nano-HA group exhibited a significantly greater reduction in lesion size at the one-month follow-up compared to the PRF and nano-HA group (P = 0.003, Effect size = 0.382). However, at three and six months, no significant differences were observed in the median percentage reduction in lesion size between the two groups (P = 0.077, Effect size = 0.092; P = 0.096, Effect size = 0.073, respectively).

## Discussion:

Periapical surgery focuses on regenerating lost bone tissue and restoring oral health. However, the healing process can be impeded by inadequate bone healing, often due to the ingrowth of non-mineralized tissue. To promote bone regeneration, biocompatible materials, including bone grafts or bone substitutes, can be utilized to fill the bony defect and create a favorable environment for bone cell proliferation and differentiation [19]. Ideal wound healing aims for maximum regeneration and minimal scarring. Tissue regeneration depends on the availability of cells and growth factors. Bioactive materials like hydroxyapatite (HA) can support bone regeneration [20]. This study assessed the effectiveness of nHA and nHA+PRF in periapical bone regeneration. Hydroxyapatite is a widely utilized bone graft material because of its structural resemblance to natural bone. A reduced particle size increases surface area, facilitating the absorption of essential proteins from blood plasma into interstices, which supports cell growth. Ideal nano-bone graft materials should possess osteoinductive properties, be entirely synthetic, have high porosity, feature a nanostructured design, be capable of absorbing protein particles within their nanoporous framework and undergo biodegradation by osteoclastic cells [21, 22, 23, 24-25^25^]. Nano hydroxyapatite (nHA), a synthetic bio ceramic with a nanoscale structure, a small size and a large surface area enhances bio reactivity [26]. Synthetic nHA bone grafts are widely used [27]. nHA's similarity to natural bone promotes osteoblast proliferation and metabolism, leading to better osseointegration and osteoconductivity. nHA can release calcium and phosphate ions, stimulating bone formation and inducing bone morphogenetic protein secretion, which helps in the recruitment and differentiation of osteoprogenitor cells into mature osteoblasts, leading to the formation of new bone tissue [28, 29-30]. Choukroun's PRF is an autologous platelet concentrate produced without anticoagulants, allowing for rapid platelet activation, which initiates coagulation and results in fibrin clot formation. The natural activation of platelets leads to the release of growth factors. PRF serves as a fibrin matrix that inherently incorporates a substantial amount of platelets and cytokines. As the fibrin gradually degrades, these components are released over time, ensuring a sustained delivery of growth factors to the wound site [31, 32, 33, 34-35]. In the present study, (PRF) gel was utilized as a bioactive scaffold to promote tissue regeneration. It is a natural, autologous biomaterial that is rich in growth factors and cytokines. These growth factors promote wound healing and tissue regeneration. The IGF-1, in particular, has been shown to stimulate bone formation by promoting the proliferation and differentiation of osteoblasts which stimulate bone formation and cell proliferation [36, 37].

The combination of Nano hydroxyapatite and platelet-rich fibrin was employed in this study to synergistically enhance bone regeneration and minimize scar tissue formation. Several studies have proved that PRF combined with nanocrystalline HA and collagen can significantly enhance bone regeneration compared to conventional techniques and PRF alone [38, 39]. Several factors contributed to the success of the surgical treatment in this study, including the use of the crown-down technique. This approach enabled direct access to the apical third, facilitated the removal of a large volume of necrotic material and bacteria before apical preparation, enhanced the penetration depth of irrigants into the lateral canals and provided greater control over the entire canal length. NaOCl was selected as the disinfectant solution due to its antimicrobial properties and tissue dissolution capabilities. Additionally, achieving an apical seal through the use of root-end filling materials is crucial to preventing the continuous ingress of bacteria and oral fluids into the periapical tissues, thereby promoting healing and reducing the risk of recurrent infection. Mineral Trioxide Aggregate (MTA) is widely regarded as the preferred retrograde filling material due to its excellent sealing properties, biocompatibility and high success rates in promoting healing [40, 41, 42, 43-44]. A bi-mix antibiotic paste was utilized as an intracanal medicament between visits, which has been investigated as a potential therapeutic strategy for intracanal medicaments [45, 46-47]. It was observed that the nano-HA group showed significant reductions in lesion size over time. While the PRF and nano HA also showed significant reductions at 1 and 1-3 months, but not at 3-6 months. Regarding the nano-HA+PRF and nano-HA groups at 1, 3 and 6 months. No significant difference was found between them. They showed a significant reduction in lesion size. The current findings align with Basta *et al.* suggesting that combining PRF with synthetic bone grafts enhances periapical healing [48]. Elbattawy *et al.* found that nHA significantly reduced clinical and radiographic parameters after 6 months, aligning with our findings [49]. Khetarpal *et al.* demonstrated that combining MTA with platelet-rich fibrin can significantly enhance healing in complex cases. Since PRF is a natural biological material derived from the patient's own blood and is rich in various growth factors, it played a vital role in expediting the healing process in this study. The synergy between MTA's biocompatibility and sealing properties and PRF's regenerative potential resulted in improved clinical outcomes and reduced healing time [50].

## Conclusion:

The properties of nHA appear to promote tissue regeneration and accelerate bone formation. The combination of nanohydroxyapatite and platelet-rich fibrin significantly enhanced periapical healing within the first three months postoperatively compared to nHA alone. The synergistic interaction between nHA and PRF, involving the release of growth factors from PRF along with the osteo-conductive properties of nHA, contributed to this accelerated healing process.

## Figures and Tables

**Table 1:** Kruskal-Wallis test results and descriptive statistics for lesion size comparison

**Time**	**Nano-Hydroxyapatite (Group 1)**		**PRF and Nano Hydroxyapatite (Group 2)**		**P-value**	**Effect size (Eta Squared)**
	**Mean (SD)**	**Median (range)**	**Mean(SD)**	**Median (range)**		
Preoperative	10.1 (4.9)	8.5 (5-19.2)	8.4 (2.5)	8.2 (5.7-12.5)	0.55	0.08
1 month	3.6 (3.8)	4.2 (0-10.5)	2.5 (3.0)	1.9 (0-7.8)	0.12	0.054
3 month	1.4 (2.0)	0 (0-5.8)	0.8 (1.2)	0 (0-3.0)	0.2	0.008
6 month	0.3 (0.6)	0 (0-1.6)	0 (0)	0 (0-0)	0.095	0.074

**Table 2:** Illustrates descriptive statistics and results of Friedman's test for comparison of lesion size (mm) at different follow-up periods within each group.

**Period**	**Group 1**		**Group 2**	
	**Mean (SD)**	**Median (range)**	**Mean(SD)**	**Median (range)**		
Preoperative	10.1 (4.9)	8.5 (5-19.2)	8.4 (2.5)	8.2 (5.7-12.5)
1 month	3.6 (3.8)	4.2 (0-10.5)	2.5 (3.0)	1.9 (0-7.8)
3 month	1.4 (2.0)	0 (0-5.8)	0.8 (1.2)	0 (0-3.0)
6 month	0.3 (0.6)	0 (0-1.6)	0 (0)	0 (0-0)
P-value	<0.001		<0.001	
Effect size	0.878		0.875	

**Table 3:** Illustrates the percentage reduction in lesion size.

**Time**	**Nano-Hydroxyapatite**		**PRF and Nano Hydroxyapatite**		**P-value**	**Effect size (Eta Squared)**
	**Mean (SD)**	**Median (range)**	**Mean(SD)**	**Median (range)**		
1 month	74.2 (25.8)	63.1 (45.2-100)	72.5 (31.4)	79.0 (27.5-100)	74.2 (25.8)	63.1 (45.2-100)
3 months	91.0 (11.9)	100 (71.0-100)	93.5 (10.9)	100 (72.3-100)	91.0 (11.9)	100 (71.0-100)
6 months	98.9 (4.5)	100 (88-100)	100 (0)	100 (100-100)	98.9 (4.5)	100 (88-100)
